# Characterization of a new small-molecule inhibitor of HDAC6 in glioblastoma

**DOI:** 10.1038/s41419-020-2586-x

**Published:** 2020-06-02

**Authors:** Jaione Auzmendi-Iriarte, Ander Saenz-Antoñanzas, Idoia Mikelez-Alonso, Estefania Carrasco-Garcia, Maitena Tellaetxe-Abete, Charles H. Lawrie, Nicolás Sampron, Aitziber L. Cortajarena, Ander Matheu

**Affiliations:** 1 Cellular Oncology group, Biodonostia Health Research Institute, San Sebastian, Spain; 2 Center for Cooperative Research in Biomaterials (CIC biomaGUNE), Basque Research and Technology Alliance (BRTA), San Sebastian, Spain; 3 CIBERfes, Carlos III Institute, Madrid, Spain; 4 Molecular Oncology group, Biodonostia Health Research Institute, San Sebastian, Spain; 5 IKERBASQUE, Basque Foundation for Science, Bilbao, Spain; 6 Radcliffe Department of Medicine, University of Oxford, Oxford, UK

**Keywords:** CNS cancer, Drug development

## Abstract

Histone deacetylase 6 (HDAC6) is an epigenetic modifier that is an attractive pharmacological target in cancer. In this work, we show that HDAC6 is elevated in glioblastoma, the most malignant and common brain tumor in adults, in which its high levels correlate with poor patient survival and is more abundant in glioma stem cell subpopulation. Moreover, we identified a new small-molecule inhibitor of HDAC6, which presents strong sensitivity for HDAC6 inhibition and exerts high cytotoxic activity, alone or in combination with temozolomide. It is also able to significantly reduce tumor growth in vivo. Transcriptomic analysis of patient-derived glioma stem cells revealed an increase in cell differentiation and cell death pathways, as well as a decrease in cell-cycle activity and cell division by the treatment with the compound. Finally, the comparison with a pan-HDAC inhibitor, Vorinostat (SAHA), or HDAC6-specific inhibitor, Tubastatin A, showed higher target specificity and antitumor activity of the new HDAC6 inhibitor. In conclusion, our data reveal the efficacy of a novel HDAC6 inhibitor in glioblastoma preclinical setting.

## Introduction

Glioblastoma Multiforme (GBM), also known as WHO grade IV malignant glioma, is the most common and malignant primary brain tumor in adults^1^. Patients diagnosed with GBM display a very poor prognosis with a median survival of around 15 months^2^, despite multimodal treatment including maximum surgical resection of the tumor, followed by radiotherapy and chemotherapy with temozolomide (TMZ)^3^. GBMs are notorious for resistance to therapy, which has been linked to genetic and cellular heterogeneity and limited drug delivery into the brain because of the presence of the blood–brain barrier^4–7^. Despite numerous efforts, the addition of compounds against specific genetic driver targets or biological hallmarks of GBM have largely failed^8^. Therefore, new molecules and targets for GBM effective treatment constitute an unmet medical need.

GBM origin and progression is associated not only to genetic alterations, but also to epigenetic modifications. In particular, the GBM epigenome presents both specific and general shifts in the histone-modification and DNA methylation landscapes^9,10^. In this regard, epigenetic alterations have become promising GBM diagnostic biomarkers and therapeutic targets since, unlike genetic mutations, the effect of epigenetic modifications might be reversible by the use of drugs that target enzymes involved in adding, removing, or signaling histone modifications and DNA methylation^9,11,12^. In this line, inhibitors of histone deacetylases (HDACs) and DNA demethylating agents have been approved for use in the therapy of hematologic malignancies, such as cutaneous T-cell lymphoma and myelodysplastic syndrome, respectively^12^. In GBM, epigenetic modifiers including inhibitors of HDACs have also shown promising results in preclinical trials^8,13^. However, the lack of specificity of some of these compounds still remains a clinical issue since high and, for some cases, active doses elicit deleterious side effects.

Histone deacetylase 6 (HDAC6) is a member of class IIb of HDAC family and it is becoming an attractive pharmacological target in cancer^14^. It is predominantly expressed in the cytoplasm and its deacetylase activity controls both cytoplasmic and nuclear functions. Therefore, it regulates the expression, function or even stability of several proteins, being its main substrate the cytoskeletal protein α-tubulin^15^. As most HDACs, HDAC6 regulates multiple biological pathways related to proliferation and development, and it is frequently deregulated in cancer, where its elevated levels promote tumor initiation and progression^16^. Indeed, unlike class I, II, and III HDACs knockout mice, homozygous HDAC6-deficient mice, which present hyperacetylated tubulin in most tissues, are viable and fertile^17^. This indicates that the use of HDAC6-specific inhibitors could be a safer and better tolerated therapeutic strategy than drugs that target additional HDAC classes or pan-HDAC inhibitors.

It has been recently shown that HDAC6 is overexpressed in a small set of tissues from GBM patients and also in glioma-cell lines^18,19^. Moreover, its genetic knockdown inhibits cell proliferation, impairs glioma stem cell (GSC) activity and sensitizes glioma cells to TMZ^19^, postulating its inhibition as a potential strategy for GBM treatment. In the present study, we characterized the expression of HDAC6 in several human glioblastoma cohorts and glioma stem cell subpopulation, and tested the effect of a recently described HDAC6-specific inhibitor^20^ in GBM.

## Materials and methods

### Patient databases and association studies

Clinical and transcriptomic data regarding control and glioma patient samples were all collected from GlioVis database (http://gliovis.bioinfo.cnio.es/)^21^. Overall, for *HDAC* expression analysis, RNAseq and microarray results were extracted from Rembrandt cohort (28 control and 219 GBM samples), TCGA cohort (4 control and 156 GBM samples), Gravendeel cohort (8 control, 24 grade II, 85 grade III and 159 grade IV glioma samples), Vital cohort (3 grade I, 3 grade II, 6 grade III and 28 grade IV glioma samples) and Donson cohort (5 astrocytoma and 21 GBM samples). For survival studies, in addition to Rembrandt and TCGA, data from GBM patients within Phillips cohort (*n* = 76) and Joo cohort (*n* = 57) were represented. Expression studies regarding these latter cohorts could not be performed due to lack of control samples. Selected cutoff points for Kaplan–Meier representations were designated by GlioVis database as optimal and included 8.48 (Rembrandt), 7.3 (Phillips) and 8.08 (Joo) for HDAC6; 10.04 (Rembrandt), 8.91 (Phillips), and 9.55 (Joo) for HDAC1. For statistical analysis, pairwise comparisons between group levels with corrections for multiple testing (*p*-values with Bonferroni correction) were used.

For association studies, transcriptomic data from TCGA cohort have been analyzed using the website ‘R2: Genomics Analysis and Visualization Platform’ (http://r2.amc.nl).

### Cell lines, cultures, and reagents

Patient-derived GNS166 and GNS179 stem cell lines, kindly provided by Dr. Steven Pollard^22^, were cultured in GSC medium consisting of DMEM/F-12 (Sigma) supplemented by N2, B27 (Fisher), glucose (Gibco), 100 U/ml penicillin, 100 µg/ml streptomycin and growth factors (20 ng/ml basic fibroblast growth factor (bFGF) and 20 ng/ml epidermal growth factor (EGF)) (Sigma). Glioma-cell lines U87-MG, U373-MG, U251-MG, A172, and T98-G were purchased from the ATCC and cultured in DMEM (Gibco), supplemented with 10% FBS (Gibco), 100 U/ml penicillin and 100 µg/ml streptomycin for monolayer cultures and in GSC medium for oncosphere studies as previously described^23^. All cells were maintained at standard conditions of 37 °C and 5% CO_2_ in humidified atmosphere. TMZ (Sigma), Vorinostat (Suberoylanilide hydroxamic acid, SAHA) (Cayman), Tubastatin A (Cayman) and our candidate compound were dissolved in DMSO. Information about structure, synthesis and characteristics of the novel HDAC inhibitor can be found in ref. ^20^. The name was changed to JOC1 for strategy purposes.

### Dose-response assay

Glioma-cell lines were seeded at a density of 1.5 × 10^3^ cells/well in 96-well plates, with six replicates per condition. After overnight incubation, increasing concentrations of JOC1, SAHA and Tubastatin A were added into each well. Cell viability was determined by MTT assay at 72 h after treatment. For this, MTT (3-(4,5-dimethyl-2-thiazolyl)-2,5-diphenyl-2H-tetrazolium bromide) reagent was added and, after a 3 h incubation at 37 °C and 5% CO_2_, media was removed and the formed crystals were resuspended in DMSO. Plates were measured at 570 nm using a spectrophotometer. Results were analyzed by GraphPad Prism software and IC_50_ values were calculated.

### Oncosphere formation assay

5 × 10^3^ U87-MG cells/well were seeded in non-treated six-well flat bottom plates, and treatment of JOC1 at 1 µM and 5 µM or vehicle was applied in GSC medium. Primary oncospheres (1^ry^ CSCs) were grown for 7 days, and after quantification, spheres were disaggregated with accutase (Gibco) and seeded for secondary oncospheres (2^ry^ CSCs) to maintain them for another 7 days. Fresh media was added every 2–3 days to the plate.

### RNA analysis

Total RNA extraction was performed by Trizol (Life Technologies). Reverse transcription was performed using random priming and Maxima First Strand cDNA Synthesis Kit (ThermoFisher), according to manufacturer’s guidelines. Quantitative real-time polymerase chain reaction (qRT-PCR) was performed by Absolute SYBR Green mix (Thermo Scientific) in a CFX384 real-time thermal cycler (BioRad). Variations in input RNA were corrected by substracting PCR threshold cycle values obtained for *GAPDH*.

### Western blot analysis

Immunoblots were performed following standard procedures^24^. Specific antibodies against HDAC6 (7558 S Cell Signalling), acetyl-α-tubulin (ab24610, Abcam), α-tubulin (ab52866, Abcam), acetyl-histone H3-lys9 (9649, Cell Signaling), histone H3 (NB500-171, Novus), PARP (ab32064, Abcam), BMI-1 (05-637, Millipore), SOX2 (AB5603, Millipore), SOX9 (AB5535, Millipore), and β-actin (A5441, Sigma) were used in the study. For secondary antibodies, horseradish peroxidase (HRP)-linked anti-rabbit (7074S, Cell Signalling) or anti-mouse (7076S, Cell Signalling) were used. Detection was performed by chemiluminiscence using NOVEX ECL Chemi Substrate (ThermoFisher).

### Immunofluorescence

Immunofluorescence was performed as described in previous studies^24^. Cells were incubated with phospho-histone H3 (p-H3) (ab14955, Abcam) and caspase-3 (AF835, R&D Systems) antibodies. Secondary antibodies anti-mouse and anti-rabbit were Alexa Fluor 555 IgG (A21422, A31572, respectively; Invitrogen). Nuclear DNA was stained with Hoechst 33342 (Sigma). Pictures were taken with an Eclipse 80i microscope and processed with the NIS Elements Advances Research software (Nikon).

### Microarray experiments and data analysis

Whole-transcriptome analysis was performed from 300 ng of RNA using *HuGene-2_0-st-v1 expression array* (Affymetrix), which covers 48,226 transcripts. Raw data were first checked for quality purposes through the Affymetrix® Expression Console™ Software v1.4.1 and TAC software v4.0. Then, data were normalized using the Robust Multi-array Average (RMA) and analyzed by Limma tool. Probesets with FDR-corrected *p*-values smaller than 0.05 were selected. Functional enrichment on Gene Ontology (GO) biological processes was performed on a smaller subset of the differentially expressed genes (corrected *p*-value < 0.001) by means of Broad Institute GSEA. Terms with an FDR-corrected *p*-value smaller than 0.05 were treated as significantly enriched. The data that support this study have been deposited in NCBI´s Gene Expression Omnibus and are accessible through GEO series accession number GSE143887.

### In vivo carcinogenesis assay

For subcutaneous xenografts, U87-MG cells were harvested with trypsin/EDTA and resuspended in PBS before injection. Tumor initiation assay was performed by injecting 3.5 × 10^5^ cells into both flanks of *Foxn1nu/Foxn1nu* nude mice (8 weeks old) and since then, mice were treated intraperitoneally with vehicle or 40 mg/kg JOC1 on a schedule of 5 days on/2 days off for 30 days (*n* = 12). Conversely, for tumor growth assay, mice were treated once tumors formed by the injection of 5 × 10^5^ cells reached 50 mm^3^. Animals were sorted into two different groups for treatment with vehicle and 50 mg/kg JOC1. In both assays, mice weight was measured daily and external calipers were used to measure tumor size twice a week. From these measurements, tumor volume was estimated by V = L × W^2^ × 0.5; where L is tumor length and W is the tumor width. JOC1 was dissolved in 10% DMSO: 35% PEG400: 55% sterile water.

### Immunohistochemistry

Tumors generated in mice were dissected, fixed in 4% formalin for 48 h and embedded in paraffin. Tumor sections were incubated with primary antibodies for Ki67 (ab15580, Abcam) or acetyl-α-tubulin (ab24610, Abcam). MACH 3 Rabbit (M3R531H) and MACH 3 Mouse HRP-Polymer (M3R530H, Biocare Medical) were used.

### Vehiculization in bovine serum albumin (BSA) nanoparticles (NPs)

BSANP@JOC1 were formulated following a coprecipitation method based on the FDA approved Paclitaxel formulation^25^. BSA (Sigma–Aldrich) (40 mg/ml) was dissolved in phosphate buffer 50 mM NaCl, 10 mM Na_2_HPO_4_ pH 11.5 (PBS-11.5) and incubated under continuous stirring at 27 °C during 5 min. Then, reduced L-Glutathione (GSH) (ThermoFisher) was added in excess at 1:80 (BSA:GSH) molar ratio to reduce the intramolecular di-sulfide bonds. This solution was incubated under stirring during 3–5 h, and then dialyzed (3.5 kDa cutoff membrane) in PBS-11.5 overnight at RT in order to eliminate the GSH. The dialyzed solution was incubated under stirring at 27 °C during 5 min and JOC1 was added at 10 mg/ml. When JOC1 was well dissolved, 96% ethanol (Scharlau) was added at 1:3 (v/v) and incubated during 10 min. Then, microparticles were eliminated by centrifugation (1.5 × 10^4^ *g*, 5 min) and the non-encapsulated JOC1 and ethanol were eliminated by dialysis (6–8 kDa cutoff membrane) in phosphate buffer at pH 7.4 (50 mM NaCl, 10 mM Na_2_HPO_4_ pH 7.4). Samples were stable during at least 1 week at RT.

### Characterization of BSANP@JOC1

The size distribution of the nanoformulation was measured by Dynamic Light Scattering (DLS) in NanoSizer (Malvern Nano‐Zs, UK) with 173° scattering angle at 25 °C directly from the final formulation in PBS pH 7.4 and the data were analyzed in Zetasizer Software 7.11. The amount of JOC1 loaded was determined after BSANP@JOC1 digestion in acetonitrile (1:19) by liquid chromatography-mass spectrometry (LC-MS) using an Ultra Performance Liquid Chromatography (UPLC) linked to a LCT XE time-of-flight mass spectrometer at the Mass Spectroscopy Platform.

### Data analysis

Results are represented as mean values ± SEM, together with the number of experiments carried out for each assay. Student’s *t*-test was used to calculate statistical significance (*p ≤ 0.05; **p ≤ 0.01; ***p ≤ 0.001). Additional tests are included in the text.

### Ethics approval

The study was approved by the Clinical Research Ethics Committee of the Donostia University Hospital (protocol AMF-EGM-2016-01) and adhered to the tenets of the Declaration of Helsinki. All processes involving animals were subjected to approval by the Research Animal Care of Biodonostia Institute.

## Results

### HDAC6 is overexpressed in human GBM samples and GSCs

**Fig. 1:**
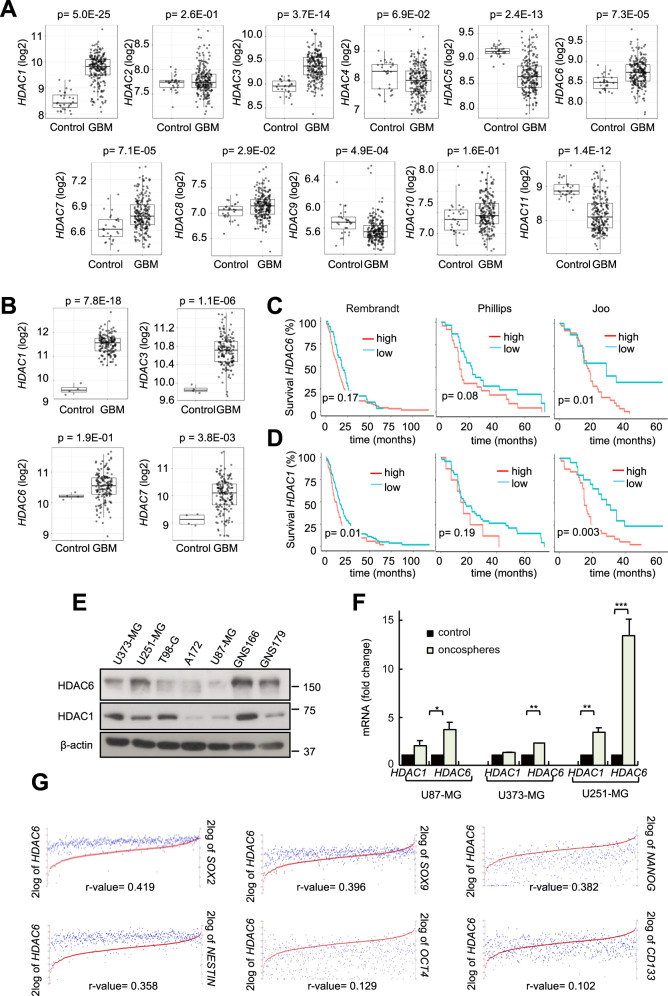
HDAC6 is overexpressed in human GBM samples and GSC subpopulation. **a** mRNA expression of the 11 human *HDAC*s in control and GBM samples from Rembrandt cohort (GlioVis: http://gliovis.bioinfo.cnio.es); **b** mRNA expression of human *HDAC1*, *3*, *6* and *7* in control and GBM samples from TCGA cohort; **c**, **d** Kaplan–Meier curves representing survival of patients with low vs high expression of **c** *HDAC6* and **d** *HDAC1* in Rembrandt (*n* = 33 vs *n* = 139; *n* = *121* vs *n* = 51; respectively), Phillips (*n* = 26 vs *n* = 24; *n* = 42 vs *n* = 8; respectively), and Joo cohorts (*n* = 10 vs *n* = 35; *n* = 14 vs *n* = 31; respectively). Optimal cutoff points were designated by GlioVis database; **e** Representative immunoblots of HDAC1 and HDAC6 expression for a set of GBM cell lines, including patient-derived GNS166 and GNS179 stem cells (*n* = 2); **f** *HDAC1* and *HDAC6* mRNA expression in U87-MG, U373-MG and U251-MG cells cultured in serum and stem cell conditions (*n* = 4); **g** Association analysis of *HDAC6* mRNA expression with *SOX2 (p* = 2.3e-24*)*, *SOX9 (p* = 1.0e-21*), NANOG (p* = 2.99e-20*), NESTIN (p* = 9.87e-18*), OCT4 (p* = 2.77e-03*)* and *CD133* (*p* = 0.018) in TCGA cohort (R2: Genomics Analysis and Visualization Platform*:* https://r2.amc.nl).

In order to determine the expression of the 11 HDACs in human GBM, we compared their mRNA levels to control brain tissues in public available datasets from Rembrandt and TCGA cohorts comprising results from 247 and 160 samples, respectively (Gliovis website^21^, http://gliovis.bioinfo.cnio.es/). Of all HDACs studied, only *HDAC 1, 3, 6* and *7* were elevated in GBM, with particular emphasis for *HDAC1* and *HDAC6* (Fig. 1a, b and Supplementary Fig. 1a). Consequently, we studied the levels of *HDAC1* and *HDAC6* in glioma samples of different grades and associated their expression to patient survival. These analyses showed that high levels of *HDAC1* and *HDAC6* correlated with decreased survival and advanced glioma grade in Rembrandt and TCGA, as well as additional cohorts (Fig. 1c, d and Supplementary Fig. 1b–d). Next, we determined their expression in several GBM cell lines and patient-derived GSCs. Immunoblot analysis confirmed that both proteins were expressed in majority of GBM cell lines and, in particular, HDAC6 was highly expressed in GSCs (Fig. 1e). In line with this, *HDAC6* mRNA expression was significantly elevated in oncospheres compared to several GBM cell lines (Fig. 1f). These results suggest that HDAC6 is enriched in GSC population. Indeed, only *HDAC6*, not *HDAC1*, expression positively correlated with several GSC markers such as *SOX2*, *SOX9*, *CD133*, *NESTIN* and *OCT4* in samples from TCGA (Fig. 1g, Supplementary Fig. 2). Together, these results confirm that GBM displays high levels of HDAC6, which are associated to GSC population.

### The novel HDAC6 inhibitor JOC1 reduces GBM cell viability and is more selective inhibiting HDAC6 function than current HDAC inhibitors

**Fig. 2:**
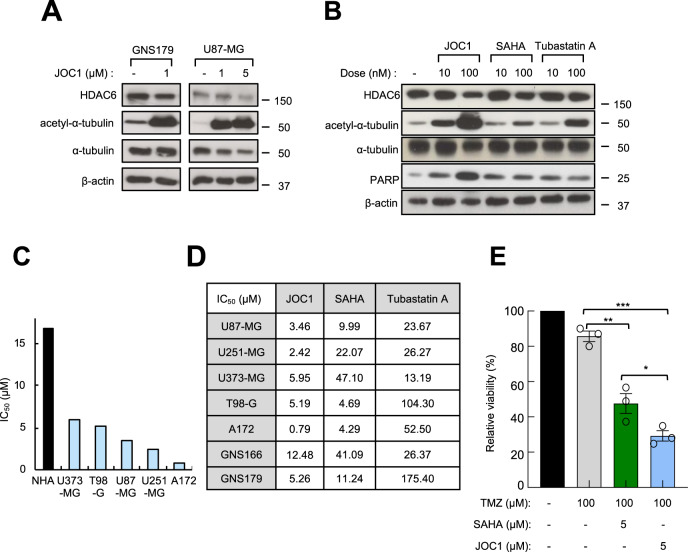
JOC1 compound reduces GBM cell viability via HDAC6 inhibition, even more efficiently than other available HDAC inhibitors. **a** Representative western blot of the effect of control, 1 µM and 5 µM JOC1 treatment for 48 h in patient-derived GNS179 and U87-MG cell line (*n* = 3) on HDAC6 and its main target (acetyl-) α-tubulin expression; **b** Western blot analysis of the expression of total HDAC6 and (acetyl-)α-tubulin in U251-MG cell line after 48 h treatment of control, 10 nM and 100 nM of JOC1, pan-inhibitor SAHA and HDAC6-selective-inhibitor Tubastatin A; **c** IC_50_ values (µM) measured by MTT assay (*n* = 3), after 72 h of increasing concentrations of JOC1 treatment in control (NHA, normal human astrocytes) and GBM cell lines; **d** IC_50_ values (µM) of JOC1, SAHA, and Tubastatin A at viability level of conventional and patient-derived GSC lines, at 72 h (*n* ≥ 3); **e** Comparative study of cell viability in combined treatments of TMZ and JOC1 or SAHA, after 72 h in U87-MG (*n* = 3).

To test the capacity of the new compound JOC1 as an HDAC6 inhibitor in GBM cells, we checked the acetylation of α-tubulin, its main target. First, we found that increasing dosage promoted higher acetylation of α-tubulin, without affecting the total expression of the enzyme, in GNS179 patient-derived and U87-MG glioma cells (Fig. 2a, b), thus providing evidence of the activity of the drug against its targeted enzyme. Next, we compared the acetylation of α-tubulin in cells treated with the pan-inhibitor SAHA, the HDAC6-selective inhibitor Tubastatin A and JOC1. Importantly, 10 nM concentration of JOC1 was able to induce α-tubulin hyperacetylation, whereas the other HDAC inhibitors did not (Fig. 2b). Moreover, 100 nM promoted stronger α-tubulin acetylation than SAHA and Tubastatin A (Fig. 2b). These results indicate the strong specificity of the new molecule in blocking HDAC6 activity in GBM cells. Similar results were obtained in mantle cell lymphoma cells^20^, revealing the efficacy of the molecule in different tumors. Moreover, JOC1 also elevated the levels of acetylated histone H3 in GBM cells (Supplementary Fig. 3a, b). These results are in agreement with the in vitro enzymatic studies of the selectivity of the compound for inhibiting HDAC function, which demonstrated an exceptional specificity for decreasing HDAC6 enzymatic activity (IC_50_ < 1 nM), being HDAC1 the second member of the family whose activity was more inhibited, but at long distance, with an IC_50_ value of over 50 nM^20^.

In order to assess the potential as an antitumor agent in GBM, we studied cell viability in a set of five independent GBM cell lines, two patient-derived GSC cultures and normal human astrocytes (NHA) treated with increasing doses for 72 h. GBM cell IC_50_ values ranged between 0.79 and 12.48 µM whereas for NHA cells was 16.85 (Fig. 2c, d), indicating that JOC1-related cytotoxic effect is higher in GBM cells than in control astrocytes. Interestingly, the comparison with SAHA and Tubastatin A showed that JOC1 presented a greater cytotoxic effect in all the seven GBM cell cultures compared to both inhibitors (Fig. 2d). In line with this, JOC1 also promoted higher induction of apoptosis, measured by cleaved PARP (Fig. 2b). Next, we tested its capacity to synergize with TMZ, studying cell viability of U87-MG cells in the presence of combined treatments of both of them. Of note, combined therapies of JOC1 or SAHA plus TMZ (5 and 100 μM, respectively) presented increased cytotoxic activity than TMZ alone, having the combination of JOC1 plus TMZ the strongest effect (30% viability vs 50% SAHA/TMZ and 85% TMZ alone) (Fig. 2e). Indeed, GBM cells are resistant to radiotherapy, and in this sense, *CHK1* reduction improves their radio-sensitivity^26^. Herein we found that JOC1 treatment reduced significantly *CHK1* expression in GNS179 and U87-MG cells (Supplementary Fig. 4a). In summary, these results confirm a robust antitumor activity of the new molecule in GBM cells.

### The novel HDAC6 inhibitor JOC1 suppresses GSC activity in vitro

**Fig. 3:**
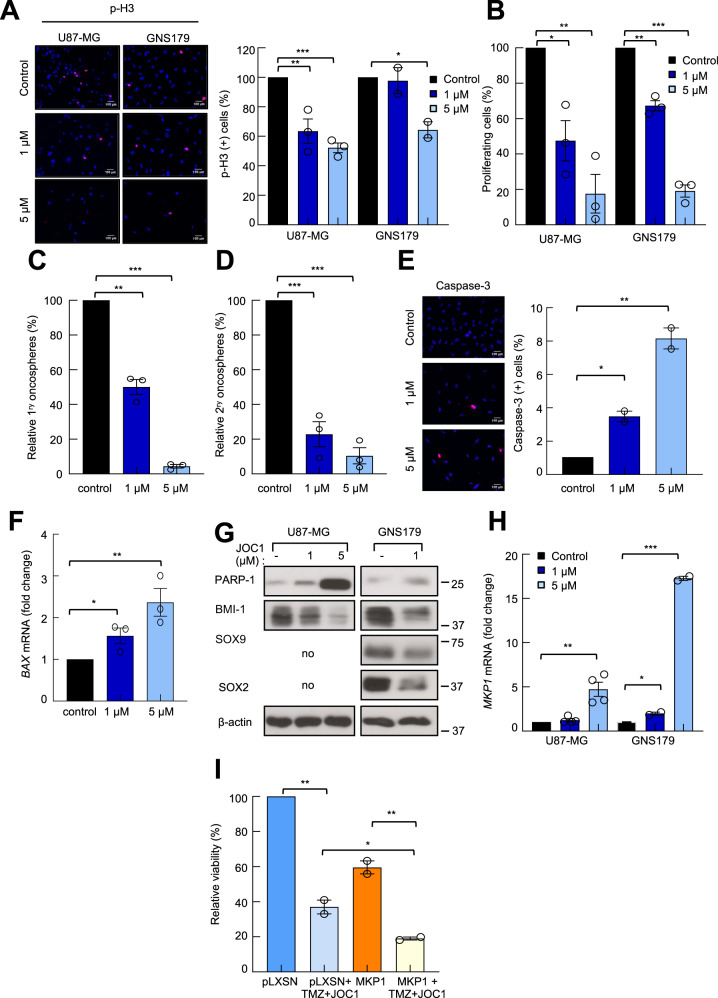
JOC1 reduces GSC proliferation and self-renewal, and induces apoptosis. **a** Representative images and quantification of p-H3^+^ cells with increasing dosage of JOC1 in both U87-MG and GNS179 cells, at 48 h (*n* = 3); **b** Proliferating cell percentage comparing U87-MG and GNS179 cells after control, 1 μM and 5 μM JOC1 72 h treatment (*n* = 3); **c**, **d** Relative quantification of 1^ry^ and 2^ry^ oncospheres forming capacity with increasing dosage of JOC1, in U87-MG cells (*n* = 3); **e** Representative images and quantification of Caspase-3^+^ cells after 48 h of control, 1 μM or 5 μM JOC1 in GNS179 cells (*n* = 2); **f** *BAX* mRNA expression in GNS179 after increasing dosage of JOC1 treatment for 48 h (*n* = 3); **g** Western blot assay of cleaved PARP-1, BMI-1, SOX9, and SOX2 after 48 h with control, 1 µM or 5 μM JOC1 treatment in GNS179 and U87-MG cells (*n* = 3). Endogenous SOX9 and SOX2 are very low and undetectable in U87-MG cells^23^; **h** *MKP1* mRNA expression in U87-MG (*n* = 4*)* and GNS179 (*n* = 2) cells treated with control, 1 µM and 5 µM JOC1 for 48 h; **i** MTT cell viability assay of U87-MG cells infected with empty vector pLXSN or MKP1 overexpression, treated with 100 µM TMZ and 1 µM JOC1 for 72 h (*n* = 2).

To test whether the novel HDAC6 inhibitor could target the population of GSCs, we first studied the proliferative capacity of GNS179 stem cells treated with increasing concentrations of the drug for 72 h. We measured the proliferating cells by counting the number of positive cells for the mitosis marker phospho-Histone3 (p-H3) by immunofluorescence (Fig. 3a), as well as by cell counting (Fig. 3b, Supplementary Fig. 4b). In both experiments, JOC1 reduced significantly the proliferation capacity of GNS179 cells in a dose-dependent manner. Similar results were also observed in U87-MG cells (Fig. 3a, b). This effect was accompanied by a significant reduction in the oncosphere formation ability of U87-MG cells, in the presence of increasing concentrations of JOC1. Thus, 1 and 5 μM JOC1 markedly reduced the number of primary oncospheres in a dose-dependent manner (Fig. 3c). Similarly, the number of secondary oncospheres was also decreased (only 25% and 15% in cells treated with 1 and 5 μM of JOC1 relative to non-treated) (Fig. 3d). Moreover, increasing concentrations of the compound also promoted a dose-dependent induction of apoptosis, represented by an increase of Caspase-3-positive cells and elevation of *BAX* and PARP expression in treated compared to non-treated cells (Fig. 3e–g). Together, these results support that the new molecule strongly inhibits GSC activity.

To further characterize its impact in GSCs, we studied the levels of several genes linked to quiescence and differentiation status^23,24^. Thus, the expression of SOX2, SOX9, and BMI-1 stem cell markers was reduced in treated cells (Fig. 3g), whereas the levels of the differentiation marker *MKP1* were elevated (Fig. 3h), together indicating that the new molecule modulates molecular pathways linked to GSC activity. Since induction of differentiation is a major characteristic of HDAC inhibitors, we tested the involvement of *MKP1* in JOC1 response. For this, we examined cell viability and oncosphere forming capacity of U87-MG cells transduced with control or *MKP1* overexpressing vector, treated with TMZ, JOC1 or a combination of them. In both experiments, high levels of *MKP1* sensitized GBM cells to both therapeutic strategies (Fig. 3i, Supplementary Fig. 4c), revealing that MKP1 mediates, at least in part, the activity of the new compound.

### The novel HDAC6 inhibitor JOC1 promotes differentiation and apoptosis and inhibits cell cycle in GSCs

**Fig. 4:**
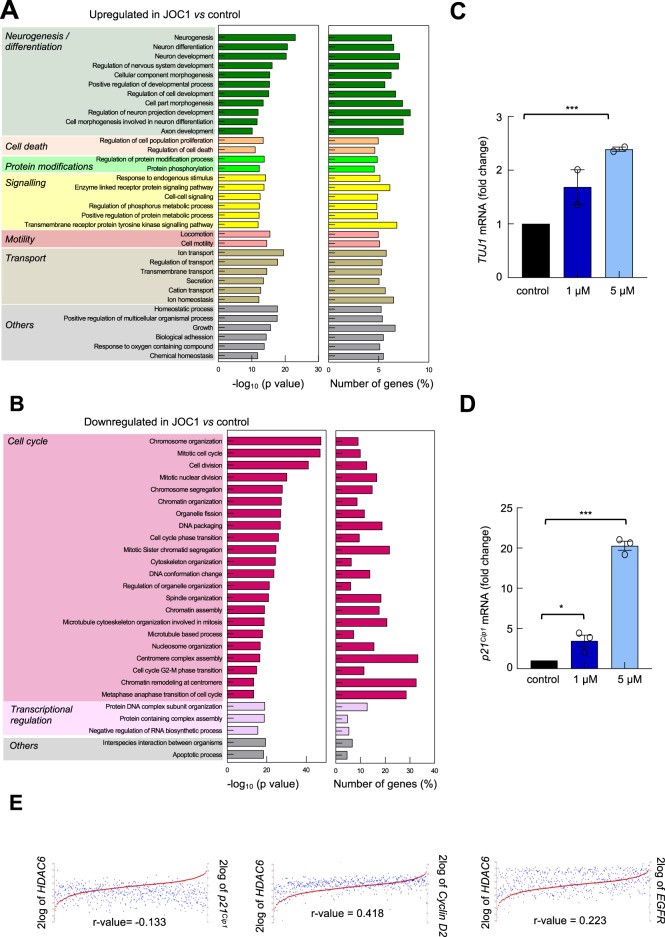
JOC1 treatment induces cell differentiation and reduces cell-cycle-associated signaling pathways. **a**, **b** Representative bar plot of biological processes upregulated and downregulated by 5 µM JOC1 in GNS166 cells (*n* = 3), after gene ontology analysis of microarray results. All genes selected from microarray study for gene ontology analysis presented fold change >1.5 and *p*-value < 0.001; **c, d** *TUJ1* and *p21*^*Cip1*^ mRNA expression in GNS179 cells treated with control, 1 µM or 5 µM JOC1 for 48 h (*n* = 3); **e** Association analysis of *HDAC6* with cell-cycle markers *p21*^*Cip1*^ (*p* = 1.97e-03), *Cyclin D2 (p* = 3.16e-24), and *EGFR (p* = 1.63e-07) in TCGA cohort (R2: Genomics Analysis and Visualization Platform*:* https://r2.amc.nl).

To identify the comprehensive profile of genes whose expression is altered by JOC1, we performed microarray gene expression analysis in GNS166 cells in the absence or presence of 5 μM of the drug. Overall, we identified over 1,000 genes whose expression was significantly changed by treatment of JOC1 (Supplementary Table 1). In order to cluster the genes altered significantly by it, we developed a gene ontology analysis, classifying them into different biological processes. Interestingly, the top canonical pathways within the upregulated networks were associated with cell differentiation and cell death (Fig. 4a), while pathways linked to cell cycle, cell division and transcriptional regulation were downregulated (Fig. 4b). Confirming the above results, *MKP1*, PARP, and acetyl-α-tubulin were elevated, whereas BMI-1 was reduced in those samples (Supplementary Fig. 5a, b). Similarly, we observed an upregulation of the neuronal marker *TUJ1* and the cell-cycle inhibitor *p21*^*Cip1*^ cells in the presence of JOC1 in GNS179 and U87-MG (Fig. 4c, d, Supplementary Fig. 5c). These results were translated into clinical data where there was a positive correlation between *HDAC6* and cell cycle and transcription regulators *p21*^*Cip1*^*, Cyclin D2, CDK11, CDK19* and *EGFR* in the TCGA cohort (Fig. 4e, Supplementary Fig. 6a). On the contrary, there was not significant correlation with almost any of the markers in the case of *HDAC1* (Supplementary Fig. 6b).

With the aim of further characterizing the difference on the efficacy of JOC1 and the pan-inhibitor SAHA, we performed the same microarray gene expression analysis for SAHA, and compare it with JOC1. Although a huge amount of altered genes were common for both drugs, JOC1 upregulated and downregulated higher number of genes (Supplementary Fig. 7a, b, Supplementary Table 1). Comparison of gene ontology analysis for both drugs confirmed the above-mentioned canonical pathways and revealed more significant differences, based on q-values, for JOC1, being cell cycle the one with higher divergences (Supplementary Fig. 7c). These data reveal advanced mechanistic insight of JOC1 functionality, which further supports its antitumor activity at molecular level.

### The novel HDAC6 inhibitor JOC1 reduces GBM tumorigenecity in vivo

**Fig. 5:**
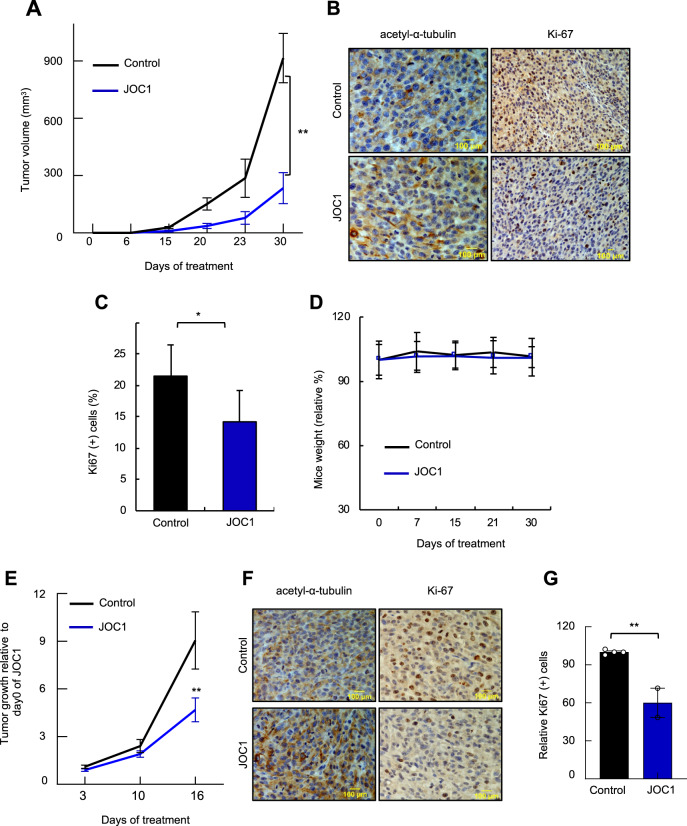
JOC1 reduces tumor initiation and tumor growth in vivo. **a** Tumor volume of mice treated with vehicle or 40 mg/kg JOC1 since cell injection, scored at the indicated time points (*n* = 12); **b** Representative images of acetyl-α-tubulin and Ki67 immunohistochemical staining from tumors obtained in **a** (*n* = 4); **c** Quantification of Ki67^+^ cells (*n* = 4); **d** Body weight changes of mice relative to their initial status; **e** Tumor growth relative to tumor volume at the beginning of JOC1 treatment at the indicated time points; **f** Representative images of acetyl-α-tubulin and Ki67 staining from tumors in **e**; **g** Relative quantification of Ki67^+^ cells in JOC1 treated compared to control tumors (*n* = 3).

To evaluate JOC1 antitumorigenic potential in vivo, we first studied its effect in tumor initiation. For that, U87-MG cells were injected subcutaneously in immunodeficient mice and they were treated with vehicle and 40 mg/kg JOC1 for 30 days intraperitoneally (Supplementary Fig. 8a). We found that the compound delayed tumor initiation and reduced tumor growth in vivo, reaching a decrease of ~80% of the final tumor volume (Fig. 5a, Supplementary Fig. 8b). It is noteworthy that tumors from treated condition presented greater acetyl-α-tubulin immunohistochemical staining and significantly less positive cells for Ki67 proliferation marker compared to non-treated ones (Fig. 5b, c), which validates the in vivo activity of the compound. Mice weight was measured as a toxicity control during the time of the experiment, and no differences were detected between both groups (Fig. 5d). Taking into account these results, we performed a more clinical in vivo assay, where the compound was administrated once the tumor was formed. In this case, we increased the dosage to 50 mg/kg for 16 days (Supplementary Fig. 8c). Of note, JOC1 reduced ~50% the volume of tumors formed in non-treated animals (Fig. 5e, Supplementary Fig. 8d), and those also presented more intense acetyl-α-tubulin staining and less Ki67-positive cells (Fig. 5f, g). Again, we did not detect differences in mice weight in this experiment (Supplementary Fig. 8e). Together, these data demonstrate that treatment with the new molecule presents potent antitumorigenic activity in vivo and seems not to alter mice weight.

### The vehiculization of novel HDAC6 inhibitor JOC1 using protein nanoparticles improves its efficacy

**Fig. 6:**
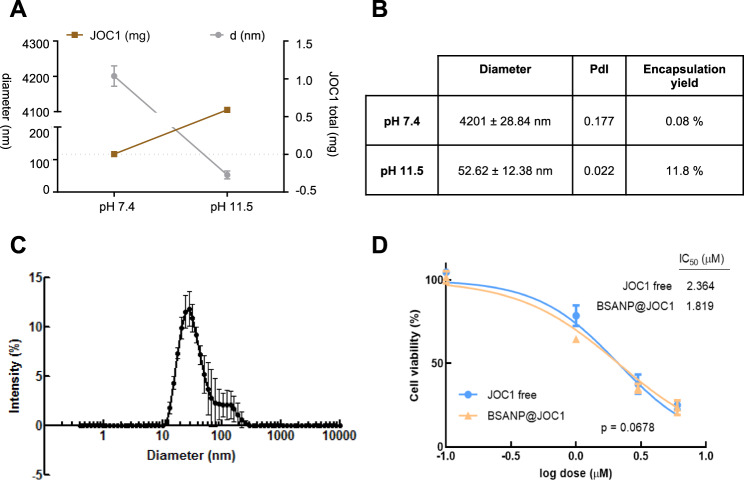
BSA-based encapsulation of JOC1 increases free-compound solubility and efficacy. **a** Representation and **b** values of encapsulation yields and size of BSANP@JOC1 at pH 7.4 and 11.5; **c** Size distribution of synthesized BSANP@JOC1, at pH 11.5 and 1 mg/ml JOC1; **d** Cell viability of U87-MG cells after 72 h of control, free JOC1 and BSANP@JOC1 treatment (*n* = 3).

Since the cross of the blood–brain barrier, and thus, its arrival into the brain is a limitation in brain tumors therapy, we designed a strategy for JOC1 vehiculization based on BSA nanoparticles (BSANP@JOC1). We evaluated the encapsulation of the drug, first by testing the solubility of the molecule at different pHs. At pHs above 10.5, fully transparent solution was obtained at 5 mg/ml concentration (Supplementary Fig. 9a). Then, the effect of the pH on the BSA-nanoparticles formation was evaluated by measuring the size distribution of the nanoparticles synthesized at different pH. The higher the pH, the smaller the size (Supplementary Fig. 9b), so pH 11.5 was selected as optimal for both molecule solubility and for nanoparticle formation, since resulted in small homogenous samples. Next, encapsulation yields were evaluated. For this, JOC1 was added to the reduced BSA solution (at pH 11.5) and then ethanol was added in order to form BSANP@JOC1 by coprecipitation. In agreement with solubility data, the encapsulation yield increased more than 100 times from pH 7.4 to 11.5 and the nanoparticle size decreased markedly (Fig. 6a, b). The final BSANP@JOC1 nanoformulation had a size below 100 nm (52.62 nm), which is ideal for nanodelivery applications, a homogeneous size distribution, and high encapsulation yields (11.8%), resulting in BSANP@JOC1 at ~1 mg/ml of JOC1 (Fig. 6b, c). Finally, we compared the efficacy of BSANP@JOC1 against free compound. For that, we performed 72 h dose-response assays in U87-MG cells with control and increasing dosage of the compounds, where we saw that the encapsulation showed a tendency for improving efficacy and sensibility. Indeed, BSANP@JOC1 IC_50_ was 1.81 µM compared to 2.36 µM alone (Fig. 6d). These results show the successful vehiculization of the new molecule.

## Discussion

Although HDAC6 seems to be an attractive pharmacological target in cancer, its expression has been only studied in small sets of cases for GBM so far^18,19^. Herein, we extend this analysis to several cohorts, comprising data of over 500 samples, and determine that GBM samples present overexpression of *HDAC6* and that its high expression correlates with advanced glioma grade and poor patient survival. We also show that GSCs are specially enriched in *HDAC6*, whose expression also correlates positively with several stem cell markers in GBM samples. Together, these results support that inhibition of HDAC6 might be a promising strategy for GBM therapy.

Currently available pan-HDAC inhibitor Vorinostat (SAHA) or HDAC6 inhibitors such as Tubastatin A showed strong preclinical antitumor activity in glioma^13^. In the present study, we describe a new small-molecule inhibitor of HDAC6, JOC1, that inhibits GBM cell growth and causes programmed cell death in vitro. Moreover, its potential as an anti-cancer agent is also validated in immunocompromised in vivo models, where it reduces significantly tumor initiation and growth even after tumor occurrence. Interestingly, the target specificity and antitumor activity of the new HDAC6 inhibitor is higher than Vorinostat or Tubastatin A. These results are in agreement with a recent study where this molecule showed potent specificity in blocking HDAC6 activity and strong antitumor activity in vitro and in vivo, which surpassed the efficacy of currently available HDAC6 inhibitors, in mantle cell lymploma^20^.

Current therapy for GBM consisting on radiotherapy and chemotherapy with TMZ displays little effectiveness because they eliminate proliferative cells but do not have an effect in the subpopulation of GSC, which are maintained in quiescent state for long periods. This population is also responsible for tumor recurrence as they present self-renewal potential when activated^7^. Previous studies have shown that HDAC inhibitors such as Vorinostat^27,28^ or Tubastatin A^29^ might regulate the activity of GSCs. In this work, we reveal that JOC1 is also able to target GSCs as it markedly reduces their proliferation and self-renewal capacity as well as induces their differentiation. Moreover, its combined therapy with TMZ seems to be more effective than the individual treatment of TMZ or the combination of Vorinostat-TMZ, supporting the hypothesis that JOC1 decreases the activity of GSCs, sensitizing them to TMZ, even in a greater proportion than other available HDAC inhibitors. JOC1 also reduces the expression of *CHK1*, which has been previously associated to an enhanced radio-sensitivity^26^. These results, further supported by studies describing the association between HDAC6 and chemo and radiotherapy^19,30^, suggest that our novel compound could synergize with both gold standard GBM treatments.

In a deeper molecular view, transcriptomic analysis reveals that JOC1 significantly reduces cell-cycle pathways and increases neural differentiation and cell death directly in GSCs. Validating these results, JOC1 reduces the expression of SOX2, SOX9, and BMI-1, all of them critical regulators of GSCs^31,32^. Moreover, there is a strong positive correlation between *HDAC6* and several stem cell markers and cell-cycle activators in GBM samples from the TCGA cohort translating this association into the clinic. On the contrary, JOC1 increases the expression of differentiation markers *TUJ1* and *MKP1* in a dose-dependent manner. Our current results together with a previous study from our group demonstrating that *MKP1* overexpression reduces tumorigenic properties and induces differentiation of GSCs whilst its levels are regulated by HDAC inhibitors^24^, and with another study describing a relationship between HDAC6 activity and SOX2 expression specifically in cancer stem cells^33^, postulate these genes and their underlying pathways as potential mediators of JOC1 activity. In this line, we show that *MKP1* overexpressing cells are more sensitive for treatment alone or with TMZ. Interestingly, JOC1 modifies similar cellular processes than the treatment with Vorinostat; however, its effect is stronger than the pan-inhibitor, further supporting that it surpasses the efficacy of currently available HDAC inhibitors. This might be related to the structure of the molecule that was generated to have great selectivity to HDAC6^20^.

Several clinical trials have been conducted for the study of HDAC inhibitors, mainly Vorinostat, in GBM. These have been performed either individually or in combination with standard treatments of chemotherapy and radiotherapy (https://clinicaltrials.gov/). In terms of safety issues, these compounds present several side effects, so that doses with good tolerability have limited clinical benefits^34^. Herein, we demonstrate that the new HDAC6 inhibitor presents larger cytotoxic effects in GBM cells compared to normal human astrocytes. In the same way, no differences in weight were detected in mice treated with even 50 mg/kg of the new molecule JOC1. Of note, the doses used in our study are lower or similar to concentrations of additional pan-HDAC inhibitors or specific HDAC6 inhibitors in GBM studies^27,35,36^. These results indicate that JOC1, in addition to increased target specificity and antitumor activity than currently available HDAC inhibitors, is more selective for tumor than healthy cells and does not alter general weight in a murine in vivo model.

Drug delivery to the brain still remains to be significantly difficult in GBM due to the existence of the blood–brain barrier. Nowadays most clinically used drug delivery systems are local, but this procedure may present several side effects, with very low benefits^37^. Therefore, novel vehiculization strategies are required to target this type of cancer. In this line, FDA has already approved “nab-paclitaxel” (*abraxane*®), a solvent-free human BSA-paclitaxel nanoparticle, as metastatic breast cancer treatment^38^. Since albumin could cross brain capillary endothelial cells barrier by interacting with albumin-binding proteins, such as glycoprotein 60 (gp60) and secreted protein acidic and rich in cysteine (SPARC), which are overexpressed in glioma^39^, we developed a BSA based nanoparticle vehiculization of JOC1. Interestingly, our results confirm the encapsulation of the small molecule, which increases the in vitro antitumoral properties of the novel HDAC inhibitor.

In summary, our data reveal high levels of HDAC6 in human GBM tissues and patient-derived GSCs, which correlate with lower patient survival, and describe the new small-molecule inhibitor of HDAC6 JOC1 that inhibits GBM cell growth and GSC activity and causes programmed cell death in vitro. Moreover, its potential as an anti-cancer agent is also validated in immunocompromised in vivo models, where it reduces significantly tumor initiation and growth even after tumor occurrence. Interestingly, the target specificity and antitumor activity of the novel HDAC6 inhibitor is higher than currently available pan-HDAC or specific HDAC6 inhibitors.

## Supplementary information

Sup Fig 1

Sup Fig 2

Sup Fig 3

Sup Fig 4

Sup Fig 5

Sup Fig 6

Sup Fig 7

Sup Fig 8

Sup Fig 9

Sup Fig legends

Table 1
