# Supplementary figures and images for: Characterization of a new small-molecule inhibitor of HDAC6 in glioblastoma
Source: Cell Death Dis. 2020 Jun 2;11(6):417. doi: 10.1038/s41419-020-2586-x (PMC7265429; doi:10.1038/s41419-020-2586-x)

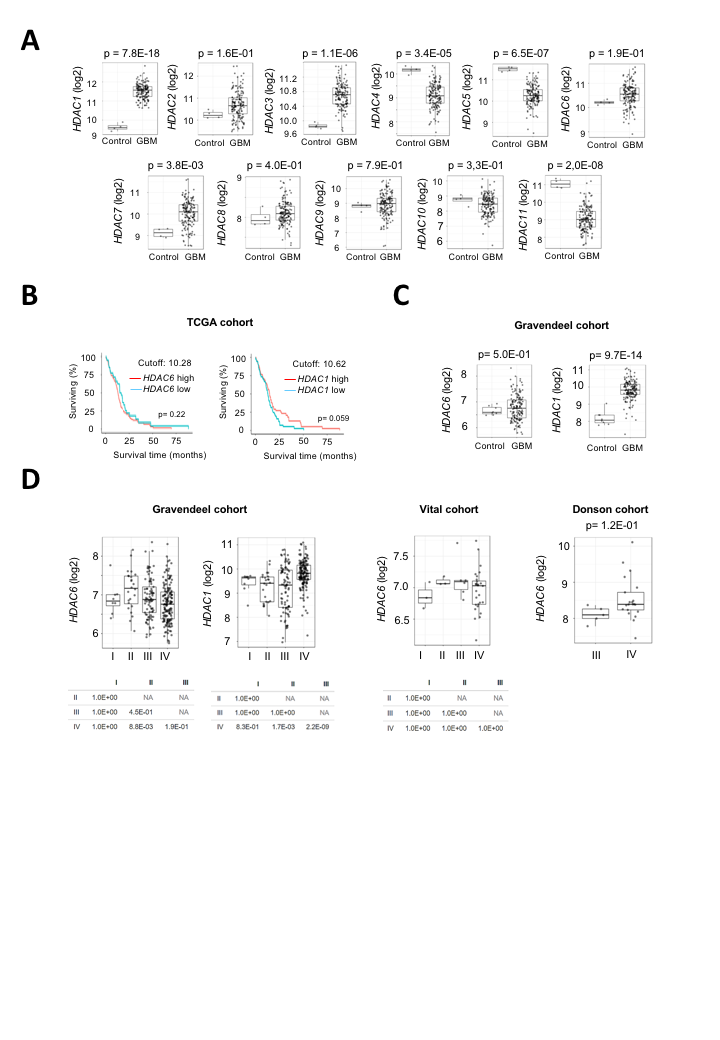

Supplement: Supplementary file 1 — Sup Fig 1 [file 41419_2020_2586_MOESM1_ESM.tif]

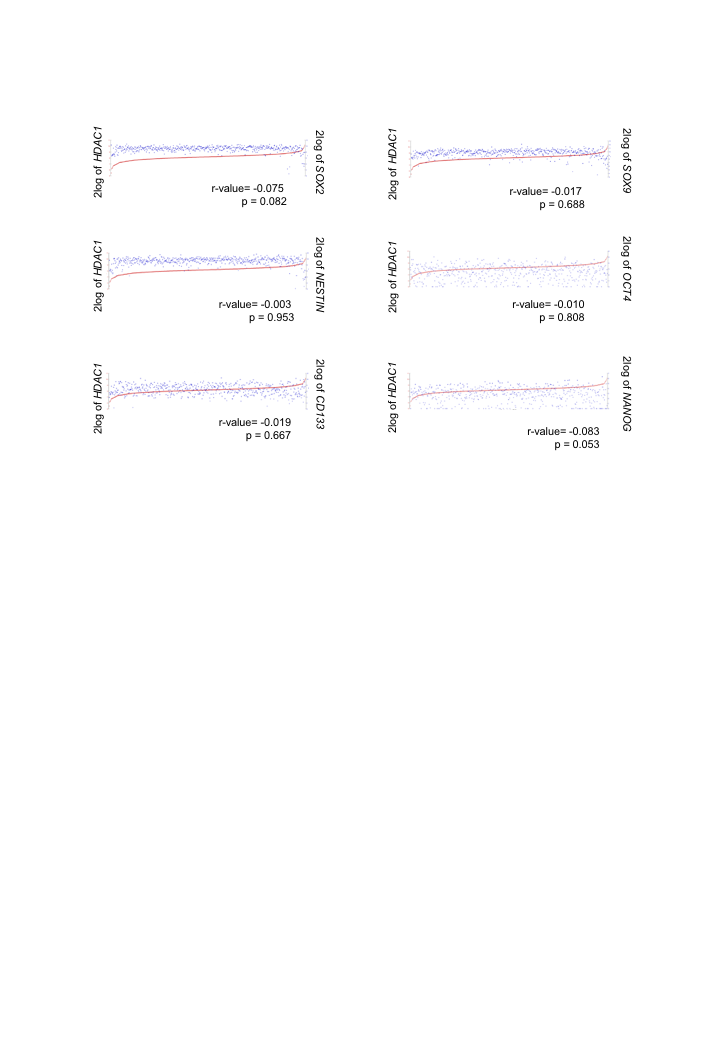

Supplement: Supplementary file 2 — Sup Fig 2 [file 41419_2020_2586_MOESM2_ESM.tif]

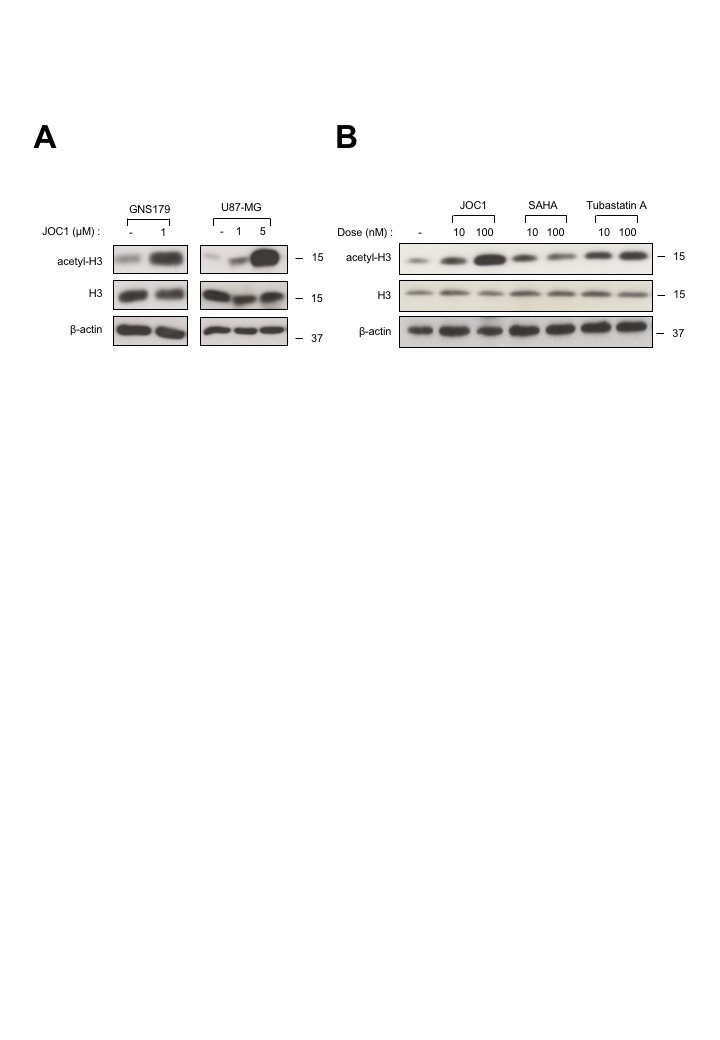

Supplement: Supplementary file 3 — Sup Fig 3 [file 41419_2020_2586_MOESM3_ESM.tif]

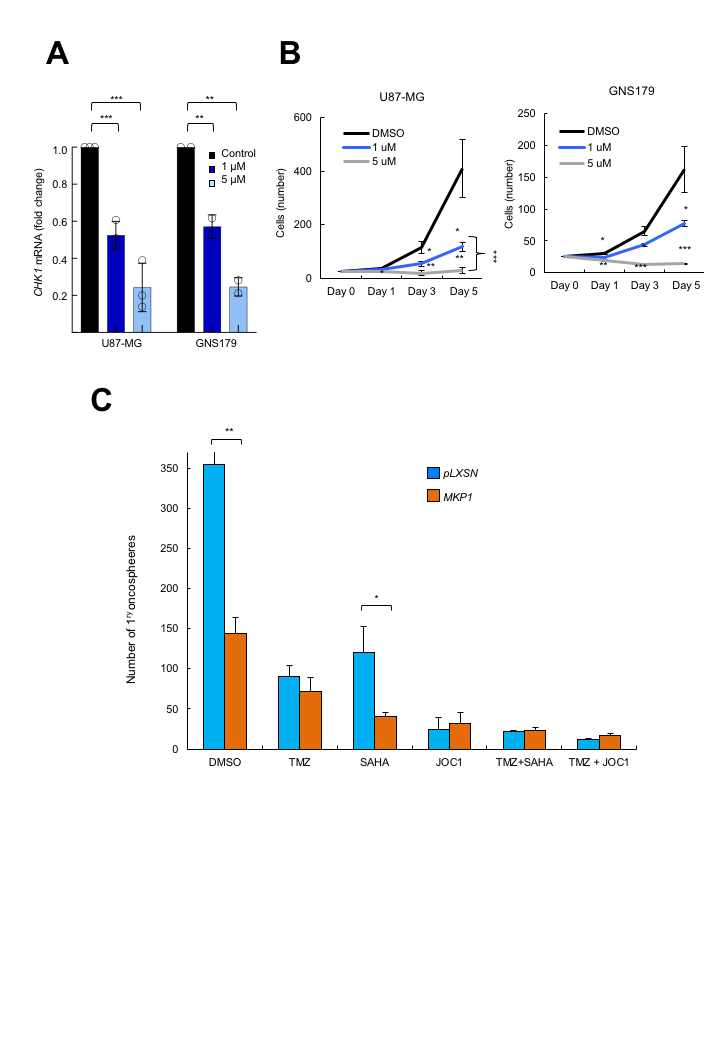

Supplement: Supplementary file 4 — Sup Fig 4 [file 41419_2020_2586_MOESM4_ESM.tif]

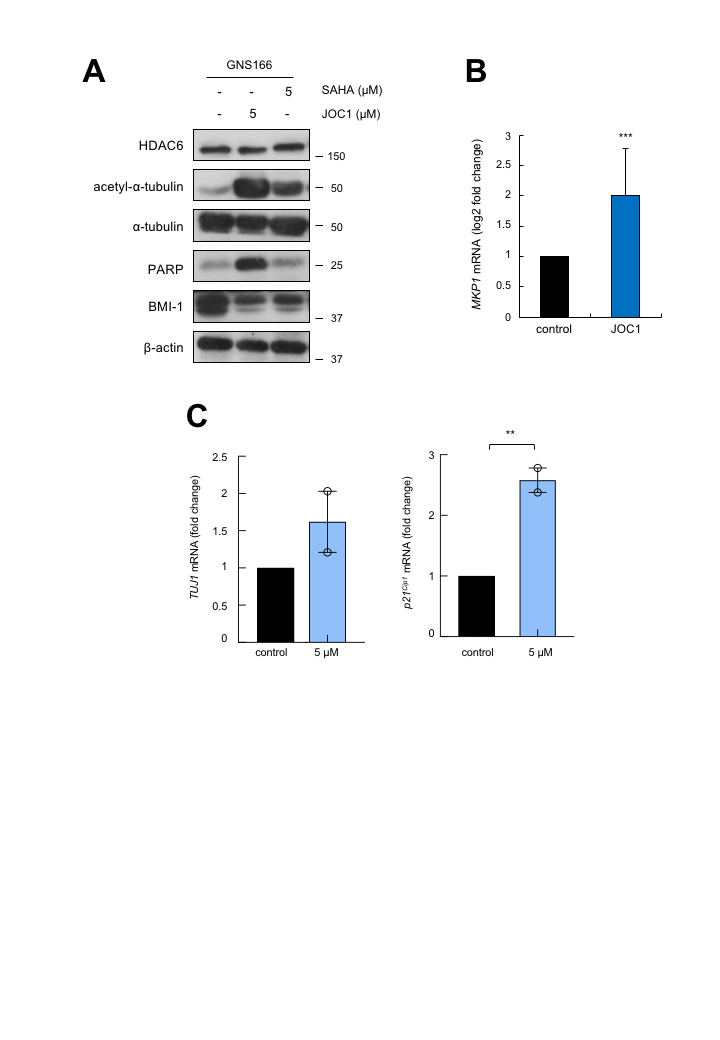

Supplement: Supplementary file 5 — Sup Fig 5 [file 41419_2020_2586_MOESM5_ESM.tif]

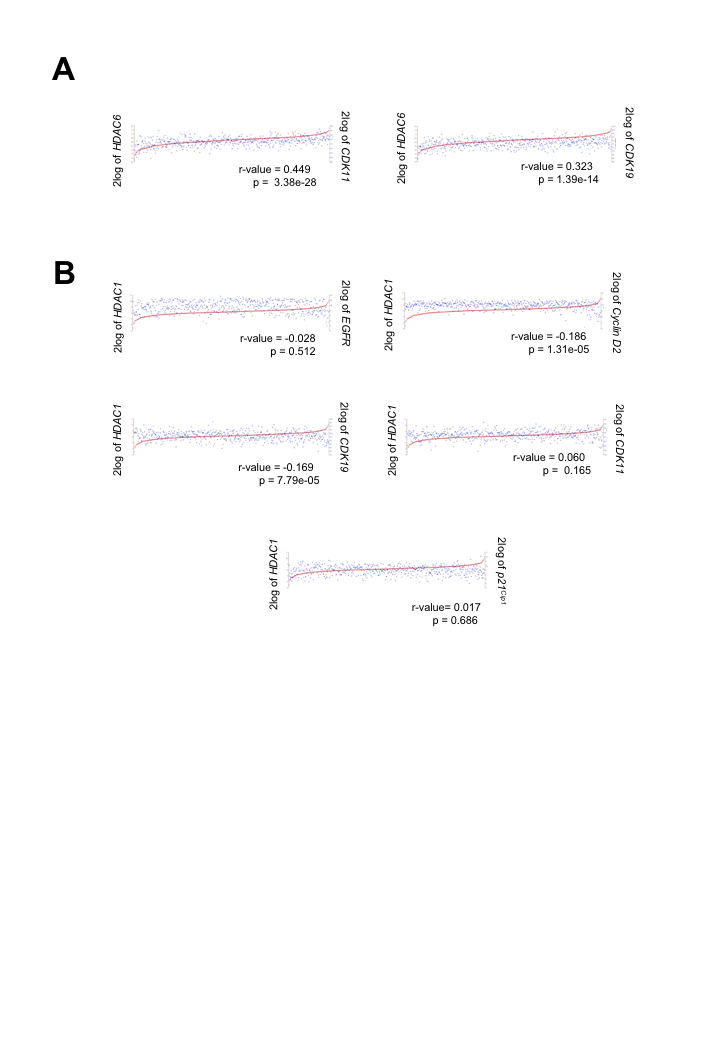

Supplement: Supplementary file 6 — Sup Fig 6 [file 41419_2020_2586_MOESM6_ESM.tif]

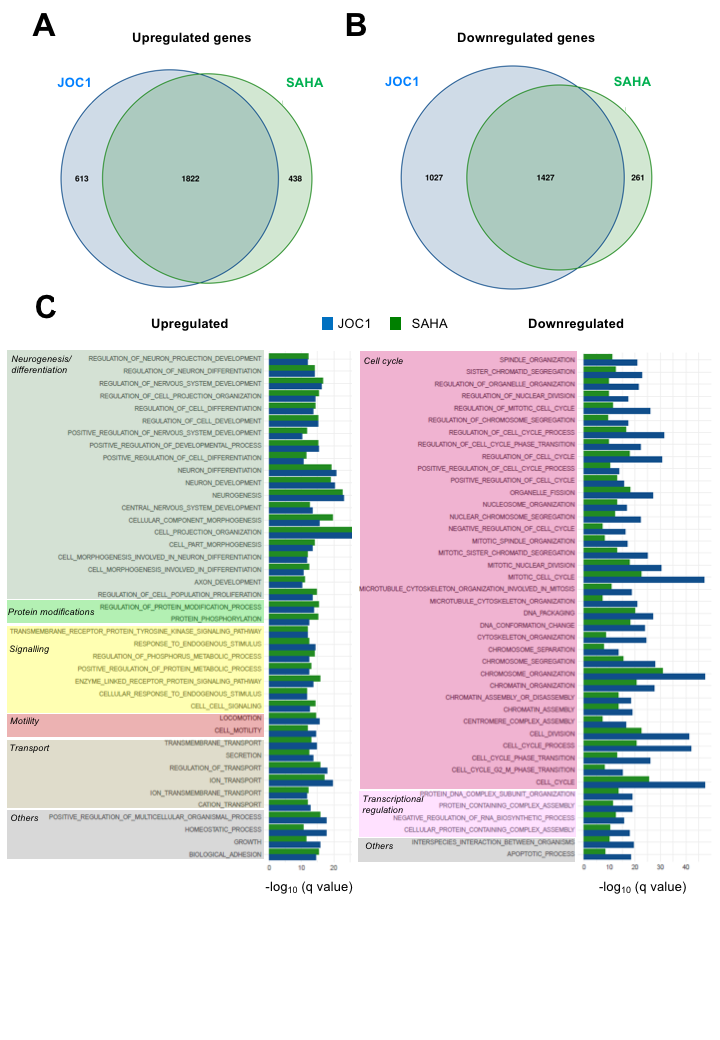

Supplement: Supplementary file 7 — Sup Fig 7 [file 41419_2020_2586_MOESM7_ESM.tif]

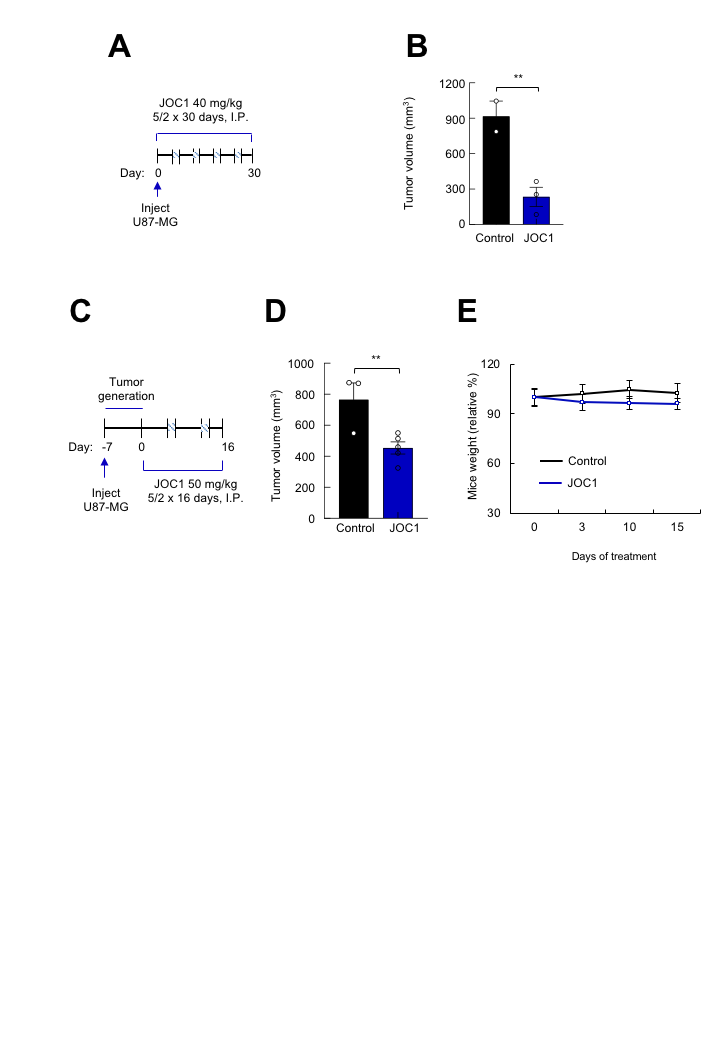

Supplement: Supplementary file 8 — Sup Fig 8 [file 41419_2020_2586_MOESM8_ESM.tif]

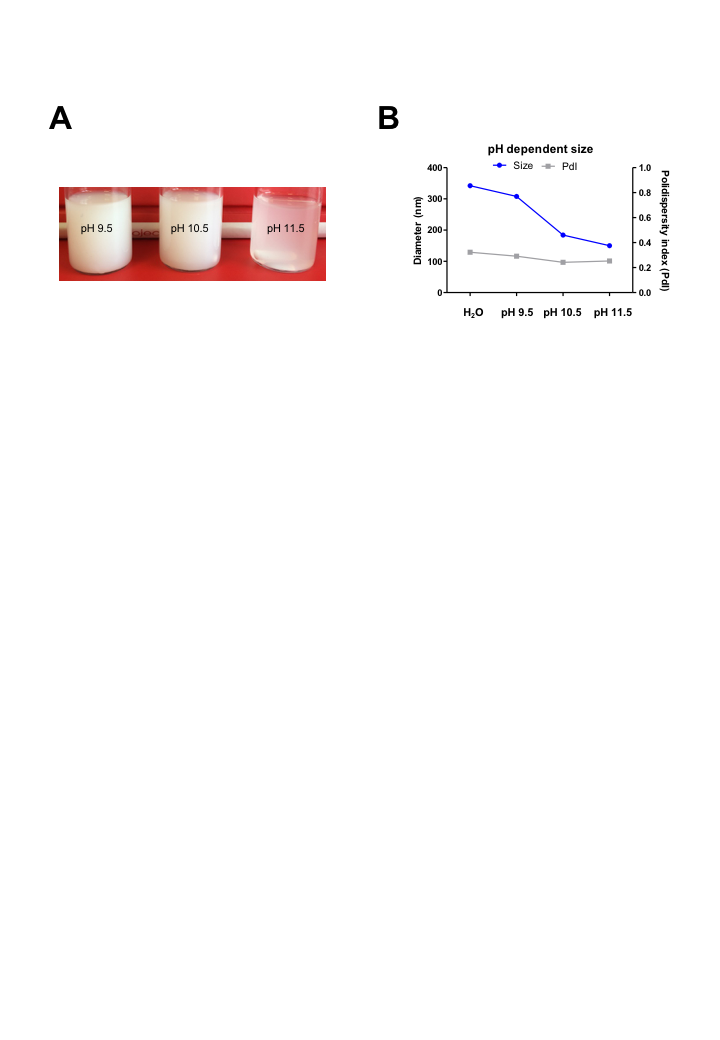

Supplement: Supplementary file 9 — Sup Fig 9 [file 41419_2020_2586_MOESM9_ESM.tif]
