# Supplementary material for: Characterization of a new small-molecule inhibitor of HDAC6 in glioblastoma
Source: Cell Death Dis. 2020 Jun 2;11(6):417. doi: 10.1038/s41419-020-2586-x (PMC7265429; doi:10.1038/s41419-020-2586-x)
Supplement: Supplementary file 10 — Sup Fig legends [file 41419_2020_2586_MOESM10_ESM.docx]

**FIGURE LEGENDS SUPPLEMENTARY**

**Suppl. Fig. 1. *HDAC6* and *HDAC1* are upregulated in glioblastoma and correlate with poor prognosis. A)** mRNA levels of the human 11 *HDACs* of control and GBM samples from RNAseq results from TCGA cohort (n= 160); **B)** Kaplan-Meier curves representing the survival of patients with low v.s. high expression of *HDAC6* (left, n= 31, n= 92, respectively) and *HDAC1* (right, n=69, n= 54, respectively). Optimal cutoff considered by GlioVis database has been represented; **C)** mRNA levels of *HDAC1* and *HDAC6* of control and GBM samples from Gravendeel cohort (n= 284); **D)** mRNA levels of *HDAC6* and *HDAC1* in different grades of glioma from Gravendeel, Vital (n= 40) and Donson (n=26) cohorts.

**Suppl. Fig. 2. *HDAC1* does not correlate with multiple stem cell marker expression.** Association analysis of *HDAC1* with *SOX2*, *SOX9*, *NESTIN*, *OCT4*, *CD133* and *NANOG* mRNA levels in TCGA cohort (R2: Genomics Analysis and Visualization Platform: https://r2.amc.nl). Significant correlation only with *NANOG*.

**Suppl. Fig. 3. JOC1 induces the acetylation of histone H3. A)** Immunoblots representing protein expression of acetyl-H3 and H3 in GNS179 and U87-MG cell lines treated with control, 1 µM or 5 µM JOC1 for 48h (n=3); **B)** Western blot analysis of the expression of acetyl-H3 and H3 of U251-MG cell line after 48h treatment of control, 0.01 µM and 1 µM JOC1, pan-inhibitor SAHA and HDAC6-selective-inhibitor Tubastatin A .

**Suppl. Fig. 4. JOC1 diminishes *CHK1* expression, reduces cell growth and oncosphere formation ability in MKP1 overexpressing cells. A)** *CHK1* mRNA levels after 48h treatment of control, 1 µM and 5 µM JOC1 in GNS179 (n=2) and U87-MG cell lines (n=3); **B)** cell counting at indicated time points in the presence of increasing concentrations of JOC1 (n=3). **C)** Oncospheres derived from U87-MG cells transduced with empty vector (pLXSN) or *MKP1* overexpression (*MKP1*), treated with TMZ, SAHA, JOC1 or their combination (n=2).

**Suppl. Fig. 5. JOC1 treatment induces cell differentiation and cell cycle arrest in patient derived stem cells. A)** Immunoblots for the validation of samples treated with control, 5 µM SAHA or 5 µM JOC1 used for microarray analysis (n=3); **B)** Fold change (log2) of *MKP1* expression in microarray analysis, error bar represents confidence interval (n=3); **C)** *p21^Cip1^* and *TUJ1* mRNA expression in U87-MG cells treated with control or 5 µM JOC1 (n=2).

**Suppl. Fig. 6. *HDAC6* correlates positively with cell cycle markers, but not *HDAC1*. A)** Association analysis of *HDAC6* with *CDK11* and *CDK19* in TCGA cohort (R2: Genomics Analysis and Visualization Platform: [*https://r2.amc.nl*](https://r2.amc.nl/)); **B)** Association analysis of *HDAC1* with *EGFR, Cyclin D2, CDK19, CDK11 and p21^Cip^* as in A. Significant correlation only with *CDK19 and Cyclin D2*.

**Suppl. Fig. 7. JOC1 treatment presents a more potent anti-oncogenic molecular pattern than SAHA. A)** Venn diagram of genes upregulated and **B)** downregulated in DMSO *vs*JOC1, DMSO *vs*SAHA and DMSO-JOC1 *vs*DMSO-SAHA; **C)** Comparison of gene ontology analysis of upregulated and downregulated genes, for JOC1 and SAHA drugs, based on q-values (n=3).

**Suppl. Fig 8. JOC1 presents anti-oncogenic activity *in vivo*. A)** Schematic representation of tumor initiation assay. 3,5x10^5^ U87-MG cells were injected *Foxn1nu/Foxn1nu* nude mice and since then, vehicle or 40 mg/kg JOC1 treatment was applied intraperitoneally by 5 days of dosing/2 days off, for 30 days; **B)** Measurement of tumor volume at final time point of the experiment; **C)** Schematic representation of tumor growth assay. 5x10^5^ U87-MG cells were injected in nude mice and, after tumor occurrence, mice were treated with vehicle or 50 mg/kg JOC1 treatment, for 16 days; **D)** Tumor volume measured at the end of experiment; E) Body weight measurement of “tumor growth” experiment, at the indicated time points.

**Suppl. Fig. 9. pH determines the size and solubility of BSANP@JOC1. A)** Representative images of solubility of BSANP@JOC1 at the indicated pH values; **B)** Size of synthesized nanoparticles at increasing values of pH.
